# Validity and reliability of a new, short symptom rating scale in patients with persistent atrial fibrillation

**DOI:** 10.1186/1477-7525-7-65

**Published:** 2009-07-15

**Authors:** Marie Härdén, Britta Nyström, Károly Kulich, Jonas Carlsson, Ann Bengtson, Nils Edvardsson

**Affiliations:** 1 Division of Cardiology, Sahlgrenska University Hospital, Göteborg, Sweden; 2 AstraZeneca HEOR R&D, Mölndal, Sweden; 3 Sahlgrenska Academy at Sahlgrenska University Hospital, Göteborg, Sweden

## Abstract

**Background:**

Symptoms related to atrial fibrillation and their impact on health-related quality of life (HRQoL) are often evaluated in clinical trials. However, there remains a need for a properly validated instrument. We aimed to develop and validate a short symptoms scale for patients with AF.

**Methods:**

One hundred and eleven patients with a variety of symptoms related to AF were scheduled for DC cardioversion. The mean age was 67.1 ± 12.1 years, and 80% were men. The patients completed the new symptoms scale, the Toronto Symptoms Check List (SCL) and the generic Short Form 36 (SF-36) the day before the planned DC cardioversion. Compliance was excellent, with only 1 of 666 answers missing.

**Results:**

One item, 'limitations in working capability', was deleted because of a low numerical response rate, as many of the patients were retired. The internal consistency reliability of the remaining six items was 0.81 (Cronbach's α). Patients scored highest in the items of 'dyspnoea on exertion', 'limitations in daily life due to AF' and 'fatigue due to AF', with scores of 4.5, 3.3 and 4.5, respectively. There was a good correlation to all relevant SF-36 domains and to the relevant questions of the SCL. The Rasch analyses showed that the items are unidimensional and that they are clearly separated and cover an adequate range. Test-retest reliability was performed in patients who failed DC and was adequate for three of six items, >0.70.

**Conclusion:**

The psychometric characteristics of the new short symptoms scale were found to have satisfactory reliability and validity.

## Background

To date there are few disease-specific instruments that assess symptoms of atrial fibrillation (AF), and they appear to be largely unvalidated and/or lack published psychometric documentation [1-6]. The generic Short Form 36 (SF-36) has frequently been used and a host of data has been generated, some of them seemingly in conflict [7-13]. Patients with AF differ in terms of underlying co-morbidity, type of AF and their perception of symptoms during AF [14-16]. Even if symptoms exist, they may often be different from one patient to another or even from one time to another. AF may even occur without symptoms or with so few symptoms that the patient does not know or suspect that they are caused by an irregular heart rhythm [17,18]. In addition, knowledge about the actual rhythm may affect the perception of symptoms [19].

To further explore symptoms in AF, we developed a very short instrument to evaluate the symptomatology of patients before and after DC cardioversion, with the specific requirements that it should be easy to understand and to complete within an ordinary 20-minute patient visit. We describe the validation of this instrument.

## Materials and methods

### Patients

Patients with persistent AF scheduled for DC cardioversion were asked to participate in the study. Patients were not required to have a certain symptomatology or degree of symptoms but were instead recruited on a consecutive basis, based on the clinical indications for DC at the hospital. There were no prespecified exclusion criteria other than inability to understand and respond to the questions and unwillingness to participate. Patients were interviewed about their underlying heart diseases and other co-morbidities and about their medical and specific arrhythmia history. A 12-lead electrocardiogram and an echocardiography were performed. The study was carried out as a part of the quality assurance program at the clinic. Patient demographics including age, sex, working status, medical history, specific arrhythmia history and all current medication were recorded by the investigator. The study was approved by the Ethics Committee of the Sahlgrenska University Hospital and was carried out in compliance with the ethical standards set forth in the Helsinki Declaration of 2005 . All patients received verbal and written information and gave their written informed consent.

The DC cardioversion was performed in the AF outpatient clinic after the patients had received adequate anticoagulation with warfarin for at least three consecutive weeks (INR 2–3). During a short general anaesthesia with propofolol, patients received one or up to four biphasic DC shocks, starting at 200J, until sinus rhythm was restored or until failure was accepted. It was rare that more than two shocks were given to the same patient. After waking up, the patients were observed for two to three hours before they met the physician, received instructions and were discharged. They were instructed not to be very active the rest of the same and the next day, and to start gradually with their normal daily activities. Except during the DC cardioversion day, the cardioversion procedure per se did not include anything that would affect the patient and his/her symptomatology two weeks later in any other way than by the change of rhythm.

### Patient-reported outcomes instruments

Patients completed three patient-reported outcome instruments: the new symptoms scale, the Toronto AF Symptoms Check List (SCL) and the Short Form Health 36 (SF-36) [7]. The Toronto AF Symptoms Check List (SCL) [20] consists of 16 item questions about symptoms that are evaluated in terms of frequency and severity. Each item is evaluated on a 1 (never) to 5 (always) frequency scale and on a 1 (mild) to 3 (severe) severity scale. The maximum frequency score is thus 80 and the maximum severity score 48. The SCL has been validated in several languages but not as yet in Swedish.

Patients also completed SF-36, an extensively used generic questionnaire containing 36 items clustered into eight dimensions. Item scores for each dimension are coded, summed and transformed to a scale from 0 (worst possible health state measured by the questionnaire) to 100 (best possible health state). This study used the Swedish acute version of SF-36, which covers a one-week recall period. The reliability and validity of the SF-36 is well documented in many languages [7].

**Figure 1:**
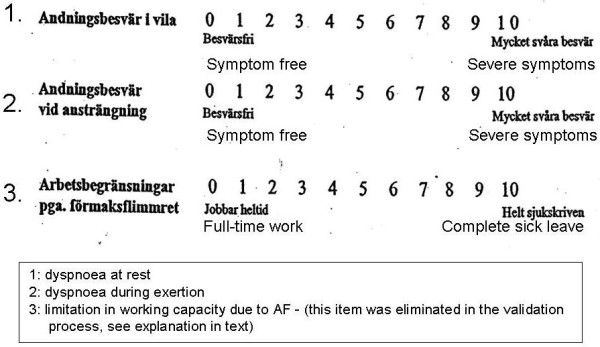
**The original items 1–3**. Item 3 was eliminated during the validation process.

**Figure 2:**
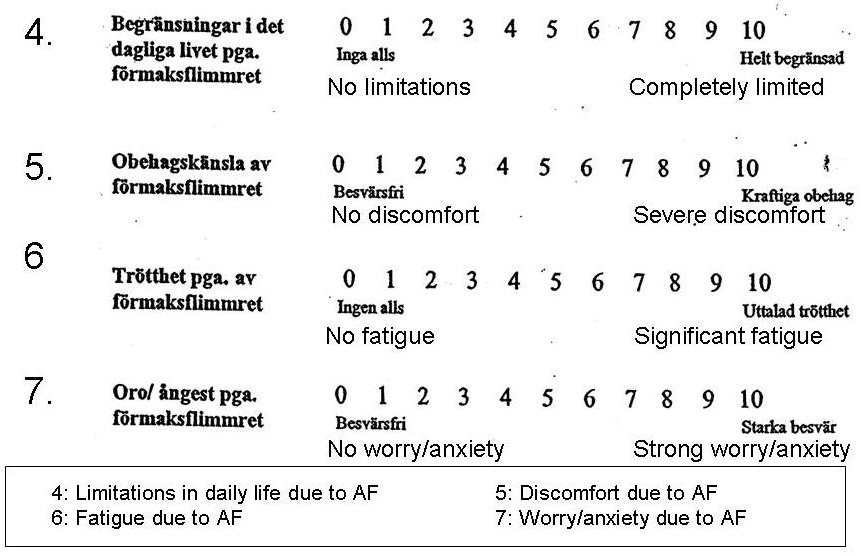
**The original items 4–7**.

The new questionnaire originally contained no more than seven questions and summarized the most frequent problems raised by patients in their contact with the nurse at the AF clinic. The item generation was based on patient interviews, and item reduction on both classical factor analysis and the modern Rasch analysis. The seven questions were the only ones ever created, and one question asking for the capacity for work was eliminated, not owing to few answers but because the answers were not numerical (Figure 1, 2).

The remaining questions focus on dyspnoea at rest and on exertion, limitations in daily life, feeling of discomfort, fatigue and worry/anxiety. Patients chose a number on a Likert scale from 0 to 10, where 0 means no and 10 severe symptoms or difficulties. The questions were formulated by the AF clinic nurse, based entirely on her clinical experience, and were all kept unchanged but for the addition of "...because of atrial fibrillation". All patients were well informed about their disease/arrhythmia and were familiar with the term "atrial fibrillation". Patients were informed about the questions and answered them without help. The questionnaire was given to them the day before and at their visit 12 ± 3 days after DC. All patients knew that they had AF at baseline, since this was confirmed with an ECG before they filled in the questionnaire. When they returned for the follow-up visit after DC, the questionnaire was given to the patient before the ECG was taken so that neither the patient nor the AF nurse knew the actual rhythm until after the questionnaire had been completed.

### Validation of the instrument

#### Reliability

Reliability was validated using measures of internal consistency (the extent to which the items are interrelated). We determined test-retest reliability (the stability of a score during serial administration of a measure by the same rater) in the nine patients who never converted to sinus rhythm. Internal consistency was assessed with Cronbach's alpha, which was calculated using data from the baseline visit. A high alpha coefficient (≥ 0.70) suggests that the items are in the same construct and support the construct validity. Test-retest reliability of symptoms was calculated in patients with AF at both the first and second visits and in whom the treatment remained unchanged. An intra-class correlation coefficient of above 0.70 indicates good test-retest reliability. This was assessed in patients who were in stable AF, with no change in treatment other than the failed DC between the two visits.

#### Construct validity

Construct validity evaluates whether the indicator actually measures the underlying attribute. The construct validity was examined by convergent, discriminant and known-groups validity using Pearson's product moment correlation. A strong correlation is considered to be over 0.60, a moderate correlation between 0.35 and 0.60 and a low correlation below 0.35 [21]. Convergent validity involves demonstrating that theoretically related dimensions of an instrument are highly correlated and was examined by correlating the items with SF-36 domains. To gain additional information, the items were also correlated with the SCL items, although the latter has not yet been validated in its Swedish translation. Discriminant validity involves showing that theoretically unrelated constructs correlate only poorly. Similar dimensions in these instruments were expected to have high correlations with each other, as shown by Pearson's product moment correlation. A strong correlation was considered to be >0.60, a moderate correlation between 0.30 and 0.60 and a low correlation <0.30 [22]. Finally, known-groups validity was used to test whether the new instrument was able to discriminate between groups of patients with different health status, in this case with severe, moderate or mild SCL symptoms.

### Statistical methods

Statistical analyses were conducted using the Statistical Analysis System (SAS version 8.02). Test results were adjusted for multiplicity (Bonferroni's correction) and reported as significant for p < 0.0001. If data were missing for more than one item in SF-36, it was substituted with the mean of the completed items in the same dimension, provided that more than half of the items in that dimension had been completed. Student's t-test was used to compare responders with non-responders with regard to the new instrument and the SCL.

## Results

In total, 137 patients were identified and eligible, 11 of whom were not interested in participating (on the grounds of a lack of time and not wishing to answer questions concerning their symptoms). One was not asked because of language problems. Fourteen patients were excluded because of nontherapeutic INR values (n = 3), pathological echocardiography (n = 1), inadequate medication (n = 1) and administrative reasons (n = 9), such as an early relapse and visit to the emergency department, intercurrent illness for other reasons leading to an emergency visit to another hospital. In addition, some patients visited another nurse than MH and did or did not receive a change in their AF treatment. They therefore did not fulfil their follow-up visit as prescribed in the protocol.

The study population thus consisted of 111 patients with a mean age of 67.1 ± 12.1 years, 89 men and 22 women. At DC, 102 (92%) patients converted to SR, 93 (84%) patients had SR at discharge and 56 (50%) of those who converted to SR remained in SR at 12 ± 3 days. Nine patients did not achieve SR at all, and nine patients relapsed within two hours. Their demographics and clinical characteristics are shown in Table 1.

**Table 1:** Baseline demographics and clinical data.

		**Sinus rhythm at 12 +- 3 days**	
	**All**	**Yes**	**No**

Patients, n	111	56	55
Age, years	67 ± 12	66 ± 11	67 ± 13
Weight, kg	86 ± 20	85 ± 17	86 ± 22
Length, cm	178 ± 9	177 ± 9	178 ± 10
BMI	27 ± 5	27 ± 5	27 ± 5
Male, n (%)	89 (80)	46 (82)	43 (78)
AF episode duration, months	5.8 ± 8.1	4.8 ± 5.4	6.9 ± 10.1
First episode of AF, n	36	20	16
Hypothyreosis, n	2 (2)	1 (2)	1 (2)
Hyperthyreosis, n	2 (2)	2 (4)	0
Hypertension, n	45 (41)	26 (46)	19 (35)
Angina pectoris, n	18 (16)	10 (18)	8 (15)
Previous myocardial infarction, n	17 (15)	7 (13)	10 (18)
Previous CABG, n	12 (11)	7 (13)	5 (9)
Previous PCI, n	9 (8)	3 (5)	6 (11)
Diabetes mellitus, n	13 (12	5 (9)	8 (15)
Heart failure, n	25 (23)	13 (23)	12 (22)
Dilated cardiomyopathy, n	11 (10)	6 (11)	5 (9)
Hypertrophic cardiomyopathy, n	3 (3)	3 (5)	0
Stroke, n	6 (5)	3 (5)	3 (5)
TIA, n	7 (6)	0	7 (13)
Venous thrombosis, n	1 (1)	1 (2)	0
Pulmonary embolism, n	1 (1)	1 (2)	0
Peripheral arterial embolism, n	1 (1)	1 (2)	0

Mean ± SD, n, % within parenthesis. BMI = body mass index;

CABG = coronary artery bypass surgery; PCI = percutaneous coronary intervention; TIA = transient ischemic attack.

The originally seven items frequently correlated strongly or moderately well with the Toronto Symptoms Check list items. Thus item 1 correlated with the severity of SCL items 1,7,8 and 15; item 2 correlated with SCL items 1, 7, 8, 11, 15; item 4 correlated with SCL items 1, 7, 8, 11, 13 and 16; item 5 correlated with SCL items 1, 2, 3, 6, 7, 9, 10, 11 and 13; item 6 correlated with SCL items 1, 2, 3, 7, 8, 11, 13 and 15; and item 7 correlated with SCL items 1, 2, 6, 8 and 10. Only item 3 did not correlate with any of the SCL severity items. The correlation with the frequency of SCL symptoms was similarly good. Item 3, 'limitations in working capability', was subsequently deleted because of a low numerical response rate, as many of the patients were retired, leaving the remaining six items to form the AF6 instrument.

Patients who achieved SR and maintained SR at the follow-up visit were defined as responders. They had a higher SCL score at baseline, 14.5 ± 7.7, and a higher severity score, 12.2 ± 7.3, than patients who did not maintain SR at follow-up (non-responders), who had 10.8 ± 7.3 and 8.6 ± 5.8, respectively. Most symptoms were mild to moderate.

At baseline, using the new instrument, non-responders scored 14 ± 9 while responders scored 22 ± 14, p = < 0.01. The highest mean item scores at baseline were 4.5 (range 0–10) for item 2, 'dyspnoea on exertion', and item 6, 'fatigue due of AF'. Item 4, 'limitations in daily life due to AF', scored 3.3 (range 0 – 10), and item 5, 'discomfort due to AF' 2.5 (range 0–10).

### Internal consistency reliability and test-retest reliability

The internal consistency of the six items forming the final AF6 instrument, measured using Cronbach's alpha, was high (0.81). Using intra-class correlation coefficients (ICC), the test-retest reliability of items varied and were highest for item 1, "dyspnoea at rest", 0.82, item 2, "dyspnoea on exertion", 0.88, and item 7, "worry/anxiety due to AF", 0.93. The ICC was low, 0.04–0.39, in item 4, 'limitations in daily life due to AF', item 5, 'discomfort of AF' and item 6, 'fatigue due to AF' (Table 2). Test-retest was performed in patients (n = 9) who were in AF on two successive occasions 12 ± 3 days apart, with only a failed DC between these times. Test-retest reliability was no better for the SF-36 domains, where four domains failed to reach an ICC of at least 0.70.

**Table 2:** Test-retest results presented as the Intraclass Correlation Coefficient, ICC, for the 6 items of the new symptoms scale AF6 and for the SF-36 domains.

	**ICC (AF at 12 ± 3 days)**	**Lower 95% CI limit for ICC**
**New symptoms scale**		
Dyspnoea at rest	0.82	0.61
Dyspnoea on exertion	0.88	0.72
Limitations in daily life due to AF	0.19	Neg
Discomfort due to AF	0.04	Neg
Fatigue due to AF	0.39	Neg
Anxiety due to AF	0.93	0.83
		
**SF-36 domains**		

Physical functioning	0.84	0.64
Role – Physical	0.56	0.17
Bodily pain	0.50	0.09
General Health	0.75	0.45
Vitality	0.77	0.49
Social functioning	0.54	0.14
Role – Emotional	0.57	0.18
Mental Health	0.95	0.10

In general, the ICC values of AF6 are lower than the SF-36 values

### Convergent and discriminant validity

The Pearson correlation coefficients used to assess the convergent and discriminant validity are shown in Table 3. Items 1 and 2 correlated strongly or moderately well with four of the eight SF-36 domains, items 5 with 5 domains, item 7 with 6 and items 4 and 6 with all eight domains.

**Table 3:** Correlation coefficients (Pearson) between the original 7 items and the SF-36 domains.

**Items**	**Dyspnoea at rest**	**Dyspnoea on exertion**	**Limitations in working**	**Limitations in daily life**	**Discomfort due to AF**	**Fatiguedue to AF**	**Anxiety due to AF**
pf_tran	-0.36437	-0.58810	-0.12466	-0.61232	-0.20312	-0.48616	-0.24267
**Physical functioning**	**< .0001**	**< .0001**	0.4315	**< .0001**	0.0325	**< .0001**	0.0106
n	111	111	42	111	111	111	110

rp_tran	-0.35569	-0.47634	-0.20069	-0.62755	-0.22794	-0.49356	-0.31813
**Role – Physical**	0.0001	**< .0001**	0.2025	**< .0001**	0.0171	**< .0001**	0.0008
n	109	109	42	108	109	109	108

bp_tran	-0.44545	-0.26582	-0.34962	-0.37650	-0.31149	-0.32431	-0.38909
**Bodily Pain**	**< .0001**	0.0048	0.0232	**< .0001**	0.0009	0.0005	**< .0001**
n	111	111	42	110	111	111	109

gh_tran	-0.29357	-0.27723	-0.29889	-0.51705	-0.31705	-0.40835	-0.38909
**General Health**	0.0019	0.0034	0.0545	**< .0001**	0.0007	**< .0001**	**< .0001**
n	110	110	42	109	110	110	109

v_tran	-0.25704	-0.40008	-0.40877	-0.66652	-0.40433	-0.66056	-0.36696
**Vitality**	0.0084	**< .0001**	0.0120	**< .0001**	**< .0001**	**< .0001**	0.0001
n	104	104	37	103	104	104	103

sf_tran	-0.14190	-0.25434	-0.08276	-0.46915	-0.38437	-0.42230	-0.37927
**Social Functioning**	0.1374	0.0071	0.6023	**< .0001**	**< .0001**	**< .0001**	**< .0001**
n	111	111	42	110	111	111	110

re-tran	-0.32767	-0.37072	0.11359	-0.52260	-0.21609	-0.48442	-0.42472
**Role – Emotional**	0.0004	**< .0001**	0.4738	**< .0001**	0.0227	**< .0001**	**< .0001**
n	111	111	42	110	111	111	103

mh_tran	-0.14611	-0.21271	-0.21615	-0.37553	-0.40701	-0.44899	-0.62826
**Mental Health**	0.1389	0.0302	0.1988	**< .0001**	**< .0001**	**< .0001**	**< .0001**
n	104	104	37	103	104	104	103

Statistically significant values (< .0001) are shown in bold figures.

### Rasch analysis

**Figure 3:**
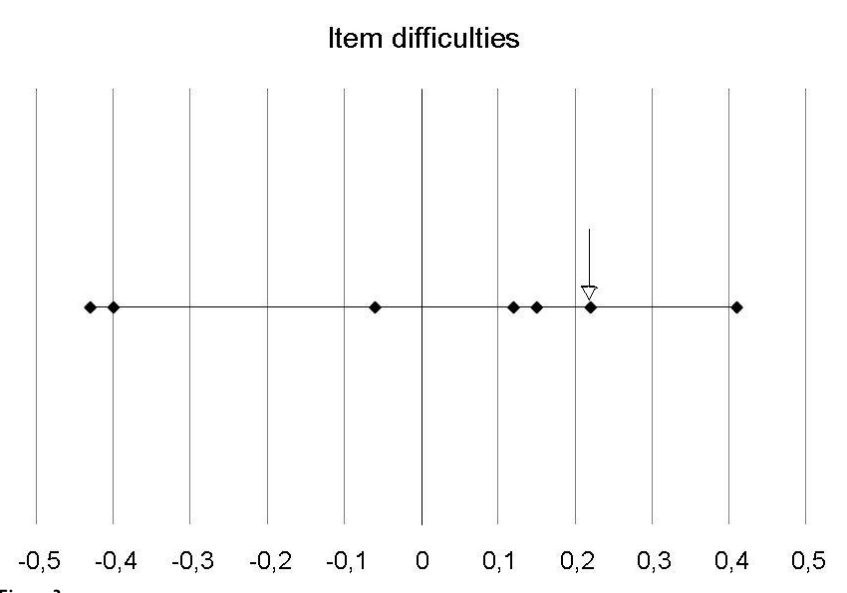
**Rasch analysis with all original 7 items included**. After removal of item 3 (arrow), the remaining items were unidimensional. The range of locations was -0.43 to +0.41.

In the Rasch analysis, each of the remaining six items were tested individually and were found to represent one domain. The range of locations was -0.43 – +0.41 (Table 4, Figure 3). In the initial analysis comprising all the original seven items, they divided themselves into two domains. When item 3 had been removed, all the remaining six items fit into one domain (based on exploratory factor analysis), thereby fulfilling the unidimensionality criterion by both factor analysis and Rasch analysis (infit – outfit statistics).

**Table 4:** Rasch analysis

**Item number**	**Raw count**	**Measure**	**Realse**	**Infit**		**Outfit**		**Score**
				**MNSQ**	**ZSTD**	**MNSQ**	**ZSTD**	**corr.**
AF1	108	0,41	0,08	0,98	-0,1	0,99	0	0,51
AF3	40	0,22	0,17	0,89	-0,4	1,28	0,4	0,39
AF7	107	0,15	0,05	1,04	0,2	1,01	0	0,65
AF5	108	0,12	0,05	1,06	0,4	1	0	0,66
AF4	107	-0,06	0,05	0,84	-1,2	0,79	-1,1	0,76
AF2	108	-0,4	0,05	1,24	1,7	1,2	1,2	0,69
AF6	108	-0,43	0,05	0,87	-1	0,84	-1,2	0,78

Mean	98	0	0,07	0,99	-0,1	1,01	-0,1	
SD	24	0,29	0,04	0,13	0,9	0,16	0,8	

### Known-groups validity

All items at baseline were compared against the three levels of symptom severity obtained from the SCL. Thus, "no symptoms" and "mild", "moderate" and "severe" symptoms were reflected in low to high item scores.

## Discussion

At the time of item generation, there was an ongoing discussion as to the value of a rhythm control versus a rate control strategy [23,24], and the AF nurse knew that there was no available validated disease-specific instrument to assess symptoms in patients with AF. In the aftermath of AFFIRM and RACE, there was a tendency towards fewer elective DCs. The items were created in the environment of patients being assessed for DC and patients being evaluated for pharmacological versus non-pharmacological treatment, and the idea was to develop a short instrument that was easy to understand and could be completed in a 20-minute visit with the AF nurse.

It was well known that patients with AF may experience anything from severe symptoms to not knowing that they have AF [16,19]. Adding to the problems, there is a well-known poor correlation with symptoms and documented AF episodes [18]. The perceptions of the patients vary and can be influenced, e.g. so that patients who know or believe that they are in SR are less symptomatic than those who know or believe that are in AF [19]. Thus, great detail in questions about symptoms or symptomatology would necessitate a very long instrument that would have to include questions that are not representative of the majority of patients with AF. Few questions were therefore created, and they were limited to ones that summarize the most common comments that patients make to the AF nurse.

In a recent publication, the validation of a new quality of life instrument started in the opposite, more traditional, way, involving item generation with the help of "AF experts" and a literature search [5]. The selection of items started at 286 expressions identified at interviews with 17 patients with AF. They were reduced to 40 after assessing "good observer/expert consistency". A further reduction could be made after administering the instrument to 112 patients with paroxysmal or persistent AF. Two factors were identified, consisting of 21 and 19 items. Following Rasch analysis, the factors were reduced to seven and 11 items, respectively, adding to the AF-QoL-18. A simple symptoms assessment scale to be used at bedside was suggested in another publication, largely a reduction of the Toronto Symptoms Severity Scale. It had however not been validated at the time of publication [2].

It is commonly accepted that patients with AF have a lower quality of life as a result of their symptomatology and that their quality of life can be improved as a consequence of treatment, measured primarily by the generic SF-36 [25]. It is also well established that at least some of that improvement is associated with the restoration and maintenance of sinus rhythm. Nevertheless, the effect of treatments has been reported to be very different in different studies. One major reason is that trials rarely require patients to have a certain degree of symptoms or symptomatology. Thus, studies evaluating the effects of catheter ablation in highly symptomatic, drug refractory patients consistently show an improvement over time that seems to be correlated to the restoration and maintenance of sinus rhythm and is also associated with objective measures, such as improvement in left ventricular ejection fraction and a decrease in left atrial dimensions. In contrast, trials comparing rhythm and rate control strategies on an intention to treat basis have failed to show any benefits of a rhythm control strategy in the patient's HRQoL. In AFFIRM, HRQoL was a predefined secondary endpoint, evaluating the perceived health "in general", using the generic SF-36 instrument at a four-week recall [8]. This and other similar trials included patients with risk factors for stroke or death who were not required to have any substantial AF or any symptoms; in addition, a considerable proportion of patients were in sinus rhythm in the rate control arm, and vice versa, thus diluting any differences, if there had been any. On the basis of available information, it is fair to anticipate that, in patients with symptoms caused by AF, the symptoms can be reduced if the patients are converted to SR and can be kept in SR for a longer period.

The primary aim of this study was to establish the initial psychometric characteristics of the AF6 instrument. The internal consistency was satisfactory, but the test-retest reliability varied between items from excellent to low. While test-retest would be adequately assessed in patients with conditions that are very stable over time, this can not be said about patients with atrial fibrillation, who may at times be in AF and at other times in SR. The low test-retest reliability of some items suggests that symptoms of persistent AF are perceived differently from time to time and/or that their frequency and/or severity may vary considerably, even over a shorter time period. In addition, test-retest reliability could only be performed in the small group of patients who had a failed DC. Another limitation was that no overall treatment evaluation of symptoms (comparing symptoms at baseline and after treatment) was utilized.

**Figure 4:**
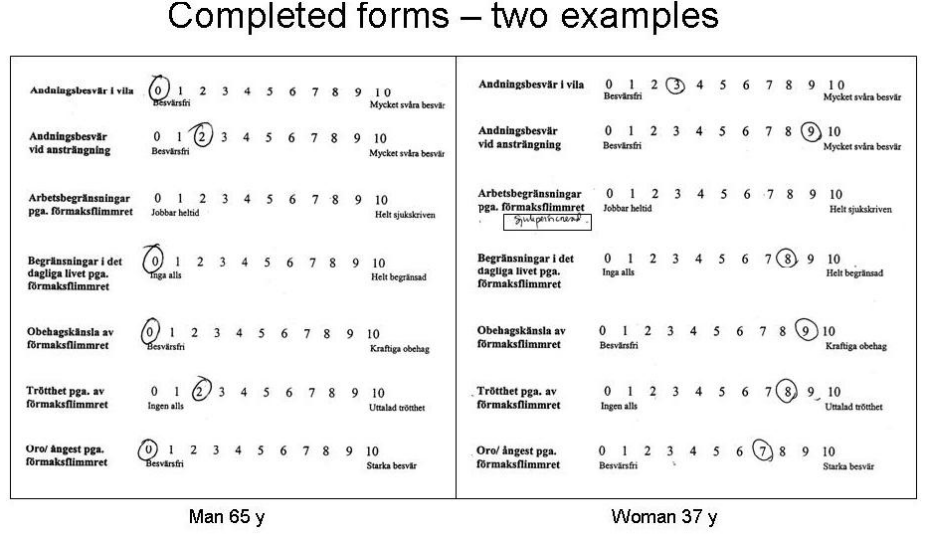
**Two examples of extremes**. Left panel: this male has few symptoms and retired from work at age 65. He felt that item 3 was no longer applicable to him and left it without a comment. Right panel: this highly symptomatic woman has complete and definite sick pension (which she indicated in hand writing instead of chosing a figure, since she felt that this was different from complete sick leave).

One important issue was that the new instrument had to be short and easy to understand. This was underlined by the excellent patient compliance. Item 3 was deleted because the question was not relevant in about half of the patients, as they were not employed (Figure 4). The remaining six items showed only one single missing response in 666 responses. Compliance in completing the SCL was equally excellent with regard to the frequency of symptoms (three missing responses in 1776 responses) and the severity of symptoms (18 missing in 1776 responses).

Known-groups validity was also proven: the AF6 instrument was able to differentiate between patients with different frequencies and severity of symptoms as documented via the SCL. We chose the patient estimation of symptomatology, since, in the case of physician estimation, much would have to be done on hearsay rather than actual symptomatology. The correlation of symptomatology and arrhythmia, especially atrial fibrillation, has also been shown to be poor. The low correlation between patient-reported and physician-assessed symptom frequency and severity indicates that symptom assessment should be balanced between the clinician's examination and the patient's report. The construct validity was also documented. Three out of the six items correlated significantly with the relevant domains of SF-36, thereby confirming its construct validity against this generic measure.

Mental state, depression, worry and anxiety seem to play important roles and may affect the relapse rate of AF as well as the symptomatology and the quality of life [26,27]. 'Anxiety due to AF' and the SF-36 mental health had the greatest impact on the patients' lives.

## Conclusion

The new short instrument is a reliable and valid instrument for assessing symptoms in patients with AF. Advantages, in addition to being AF specific, are that it can be included in a routine clinical visit, that it is easy to understand and that it had an excellent response rate. 'Dyspnoea on exertion', 'fatigue due to AF' and 'limitations in daily life due to AF' were the items with the highest scores.

## Competing interests

The authors declare that they have no competing interests.

## Authors' contributions

All the authors have read and approved the final manuscript.

MH: took part in all parts of the design and development of the AF6 and in the writing of the manuscript.

BN: participated in the data analysis, in the preparation of tables and writing of the manuscript.

KK: gave scientific input regarding the statistical methodology, the analysis of the results and in the writing of the manuscript.

JC: made statistical analyses and gave scientific input in the analysis of the results and in the writing of the manuscript.

AB: gave scientific input in the design of the study and in the writing of the manuscript.

NE: took part in all parts of the design and development of the AF6 and in the writing of the manuscript.
